# Association between gene methylation and HBV infection in hepatocellular carcinoma: A meta-analysis

**DOI:** 10.7150/jca.33005

**Published:** 2019-10-20

**Authors:** Cheng Zhang, Changxin Huang, Xinbing Sui, Xueqing Zhong, Wenjun Yang, Xiangrong Hu, Yongqiang Li

**Affiliations:** 1 Department of Medical Oncology, The Affiliated Hospital of Hangzhou Normal University, Hangzhou, Zhejiang, China; 2 Department of Gastroenterology, The Affiliated Hospital of Hangzhou Normal University, Hangzhou, Zhejiang, China; 3 Department of Pathology, The Affiliated Hospital of Hangzhou Normal University, Hangzhou, Zhejiang, China; 4 Key Laboratory of Elemene Class Anti-cancer Chinese Medicine of Zhejiang Province, Hangzhou, Zhejiang, China

**Keywords:** meta-analysis, gene methylation, hepatitis B virus, hepatocellular carcinoma

## Abstract

Gene methylation is an epigenetic alteration in hepatocellular carcinoma (HCC), and hepatitis B virus (HBV) plays a crucial role in carcinogenesis of HCC. However, the association between gene methylation and HBV infection in HCC remains unclear. In our study, we conducted a comprehensive meta-analysis to evaluate the association. A total of 1,148 studies were initially retrieved from some literature database. After a four-step filtration, we obtained 69 case-control studies in this meta-analysis. Our results showed six genes (*p16*, *RASSF1A*, *GSTP1*, *APC*, *p15* and *SFRP1*) in HBV-positive carcinoma tissues, one gene (*GSTP1*) in HBV-positive adjacent tissues and two gene (*p16* and *APC*) in HBV-positive carcinoma serums, which were significantly hypermethylated. Subgroup meta-analysis by geographical populations revealed that *GSTP1* methylation was significantly higher in HBV-positive carcinoma tissues in China and Japan. In addition, *p16* and *RASSF1A* methylation was significantly higher in China but not in Japan. Our study indicated that HBV infection could induce DNA methylation in HCC and DNA methylation could lead to the development of HBV-related HCC.

## Introduction

Primary liver cancer (PLC) is one of the most common malignant diseases, ranked sixth in incidence and fourth in mortality worldwide 1, 2. Hepatocellular carcinoma (HCC) is the most common type of PLC and it accounts for approximate 90% of PLC. HCC develops from normal liver tissues and cirrhosis through a multistep process. Hepatocarcinogenesis results from the interaction of environmental and hereditary factors, including aflatoxin exposure, alcohol abuse, genetic hemochromatosis and hepatitis virus infection 3-7. Hepatitis B virus (HBV) infection is the most prominent risk factor for the carcinogenesis of HCC, and HBV-related HCC accounts for over 80% of all HCC patients globally 8. Pathogenesis of HBV-induced HCC involves genetic and epigenetic mechanisms 9-11, especially DNA methylation 12, 13.

DNA methylation, an essential epigenetic modification, plays a crucial role in transcriptional regulation and gene expression 14-16. Methylation of the gene promoter may induce chromatin alteration to inhibit the access of the transcriptional machinery and cause gene silencing and expression decrease, thus affecting cellular biological processes 17, 18. Hypermethylation of tumor suppressor genes (TSGs) can lead to carcinogenesis of many types of carcinomas, including hepatocellular carcinoma 19, 20. In HCC, gene methylation may be enhanced by HBV infection to induce hepatocarcinogenesis 21-23. HBV-induced DNA methylation may provide a useful target for the treatment of HBV-related HCC.

Several studies also showed that geographic variation existed in DNA methylation of HBV-related HCC 19, 24. However, these studies had a small number of samples which might lead to inaccurate results. In this study, we conducted a comprehensive meta-analysis based on HBV-related HCC association studies on DNA methylation. In addition, we performed subgroup meta-analysis to evaluate DNA methylation in HBV-related HCC in geographical populations. This meta-analysis provided new clues that HBV infection could induce DNA methylation in HCC and also showed that DNA methylation could contribute to development of HBV-related HCC in some geographical populations.

## Materials and methods

### Study identification

**Figure 1:**
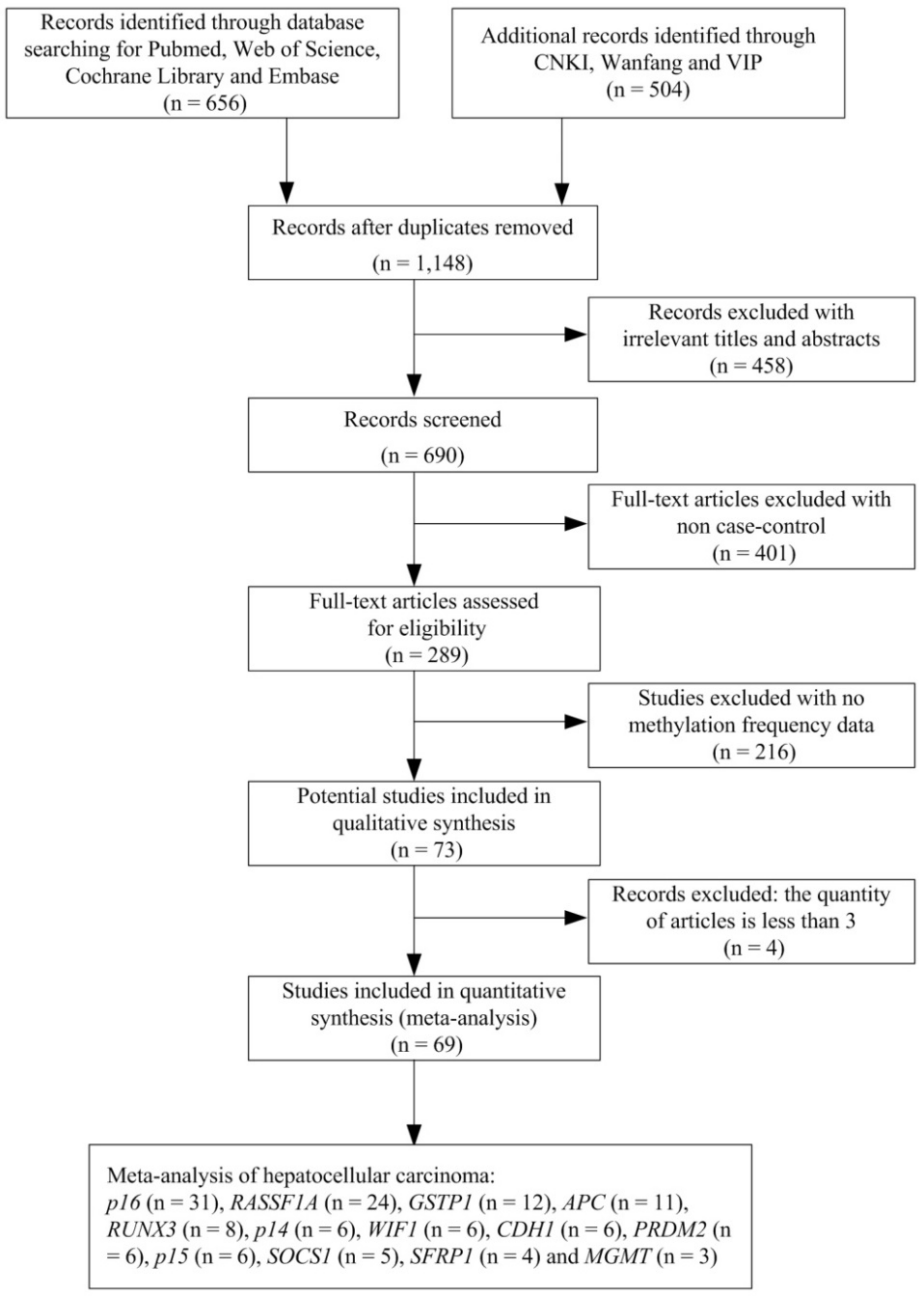
Flow diagram of the stepwise selection from the relevant studies.

We undertook a systematic review of the literature via PubMed, Web of Science, Embase, CNKI, Wanfang and VIP using the following search terms: “hepatocellular carcinoma or primary liver cancer or hepatic carcinoma or liver tumor or HCC” and “Hepatitis B virus or HBV” and “DNA methylation or gene methylation” as keywords in titles and abstracts. The search was updated until July 1, 2018. The selection of studies in this meta-analysis had to meet the following criteria: (1) they were the case-control studies with gene methylation and HBV infection in human HCC; (2) they contained the sufficient gene methylation data in order to calculate the odds ratios (ORs) and 95 % confidence intervals (CIs). We excluded the studies such as letters, books, reviews, case reports, abstracts, editorials and conference articles. Articles without case-control or methylation data were also excluded. In addition, we removed the genes with the quantity of articles less than three. The stepwise procedure of the selected studies was shown in the flow diagram of Figure 1. The meta-analysis was based on the Preferred Reporting Items for Systematic Reviews and Meta- Analysis (PRISMA) statement (Supplementary Document 1).

### Data extraction and quality assessment

Four authors (CZ, CH, XS and WY) independently applied the inclusion criteria, retrieved and extracted the data, and resolved any inconsistency through discussion with the fifth author (YL). For each included study, we extracted the following information: first author's name, publication year, sample types, HBV infection status, geographical populations and the numbers of cases and controls (Supplementary Table 1).

Four authors (CZ, CH, XS and XH) independently conducted the quality assessment, and resolved any inconsistency through discussion with the fifth author (YL). The Newcastle-Ottawa Scale was used for the methodological quality assessment of the included studies.

### Meta-analysis

The Review Manager software (version 5.2, Cochrane Collaboration, Oxford, UK) was used in the meta-analysis. The ORs and the corresponding 95% CIs were calculated and computed in the forest plots for each gene to evaluate the contribution of gene methylation and HBV infection to development of HCC. A fixed-effect model was applied for the meta-analysis with the moderate heterogeneity (I^2^ < 50%), otherwise a random-effect model was used. STATA software (version 12.0 Stata Corporation College Station, TX) was used for the meta-regression analysis to assess the potential sources of heterogeneity. Funnel plots were used to check the publication bias among the included studies. P values (p < 0.05) were considered to be significant.

## Results

A total of 1,148 studies were initially retrieved by our literature search, using the keywords “hepatocellular carcinoma or primary liver cancer or hepatic carcinoma or liver tumor or HCC” and “Hepatitis B virus or HBV” and “DNA methylation or gene methylation” from PubMed, Web of Science, Embase, CNKI, Wanfang and VIP literature database. After a series of selection procedure shown in Figure 1, we excluded 458 irrelevant studies, 401 non-case control studies, 216 studies without methylation frequency data and four studies less than three articles. Thus, a total of 69 eligible studies were included in the final meta-analysis (Supplementary Document 2). The 69 case-control studies (from 1999 to 2016) included 2,423 HBV-positive carcinoma tissues, 681 HBV-negative carcinoma tissues, 401 HBV-positive adjacent tissues, 188 HBV-negative adjacent tissues, 690 HBV-positive carcinoma serums and 172 HBV-negative carcinoma serums within 13 genes. Among these 13 genes, the meta-analysis of *p16*,* RASSF1A* and* APC* genes methylation was performed between HBV-positive carcinoma tissues and HBV-negative carcinoma tissues, HBV-positive adjacent tissues and HBV-negative adjacent tissues and finally between HBV-positive carcinoma serums and HBV-negative carcinoma serums. The meta-analysis of *GSTP1*, *RUNX3*, *SOCS1*, *CDH1* and *SFRP1* genes methylation was performed between HBV-positive carcinoma tissues and HBV-negative carcinoma tissues and between HBV-positive adjacent tissues and HBV-negative adjacent tissues. The meta-analysis of *p14*, *WIF1*, *PRDM2*, *p15* and *MGMT* gene methylation was performed between HBV-positive carcinoma tissues and HBV-negative carcinoma tissues.

**Figure 2:**
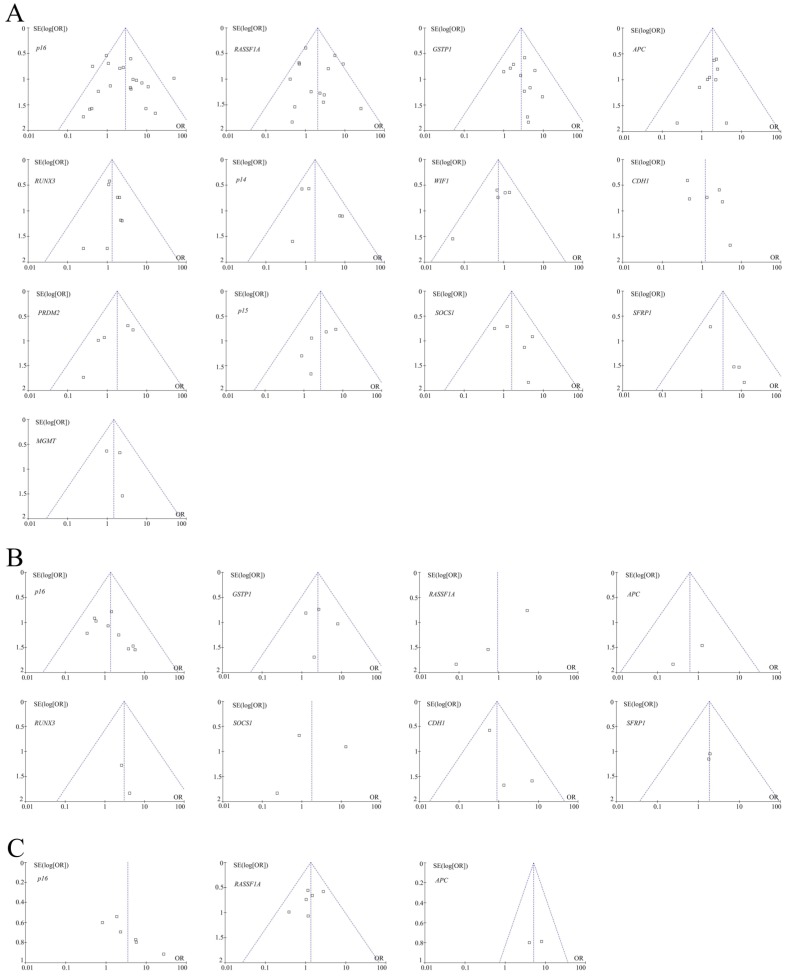
(A) Funnel plots of 13 genes methylation between HBV-positive carcinoma tissues and HBV-negative carcinoma tissues in HCC in the meta-analysis. (B) Funnel plots of eight genes methylation between HBV-positive adjacent tissues and HBV-negative adjacent tissues in HCC in the meta-analysis. (C) Funnel plots of three genes methylation between HBV-positive carcinoma serums and HBV-negative carcinoma serums in HCC in the meta-analysis.

For 13 genes reported in at least three studies between HBV-positive carcinoma tissues and HBV-negative carcinoma tissues (Table 1), no evidence of statistical heterogeneity was observed for 12 genes, including *p16* (I^2^ = 49%), *RASSF1A* (I^2^ = 46%), *GSTP1* (I^2^ = 0%), *APC* (I^2^ = 0%), *RUNX3* (I^2^ = 0%), *p14* (I^2^ = 39%), *WIF1* (I^2^ = 0%), *PRDM2* (I^2^ = 15%), *p15* (I^2^ = 0%), *SOCS1* (I^2^ = 8%), *SFRP1* (I^2^ = 0%) and *MGMT* (I^2^ = 0%). No visual bias was shown in the meta-analysis of the above 12 genes (Figure 2). Our data also demonstrated a significant heterogeneity of the remaining *CDH1* gene (I^2^ = 56%). Therefore, the random effect test was applied for the meta-analysis of *CDH1* gene. The funnel plot was shown in Figure 2.

For eight genes reported in at least three studies between HBV-positive adjacent tissues and HBV-negative adjacent tissues (Table 2), no evidence of statistical heterogeneity was observed for six genes, including *p16* (I^2^ = 0%), *GSTP1* (I^2^ = 0%), *APC* (I^2^ = 0%), *RUNX3* (I^2^ = 0%), *CDH1* (I^2^ = 11%) and *SFRP1* (I^2^ = 0%). No visual bias was shown in the meta-analysis of the above six genes (Figure 2). Our data also demonstrated a significant heterogeneity of the remaining two genes that included* RASSF1A* (I^2^ = 63%) and *SOCS1* (I^2^ = 72%). Therefore, random effect tests were applied for the meta-analysis of the above two genes. Their funnel plots were shown in Figure 2.

For three genes reported in at least three studies between HBV-positive carcinoma serums and HBV-negative carcinoma serums (Table 3), no evidence of statistical heterogeneity was observed for two genes, including *RASSF1A* (I^2^ = 0%) and *APC* (I^2^ = 0%). No visual bias was shown in the meta-analysis of the above two genes and their funnel plots were shown in Figure 2. Our data also demonstrated a significant heterogeneity of the remaining *p16* gene (I^2^ = 55%). Therefore, the random effect test was applied for the meta-analysis of *p16* gene. The funnel plot was shown in Figure 2.

As shown in Table 1, the meta-analysis of *p16* gene was involved with 24 studies between 708 HBV-positive carcinoma tissues and 224 HBV-negative carcinoma tissues. Our results revealed that the frequency of *p16* gene methylation in HBV-positive carcinoma tissues was significantly higher than HBV-negative carcinoma tissues (the overall OR = 2.87, 95% CI = 2.05-4.01, p < 0.00001). The meta-analysis of *RASSF1A* methylation between 914 HBV-positive carcinoma tissues and 228 HBV-negative carcinoma tissues indicated a statistical difference (the overall OR = 2.05, 95% CI = 1.44-2.92, p < 0.0001). The same consequence was also found in the other four genes including *GSTP1* (the overall OR = 2.50, 95% CI = 1.52-4.12, p = 0.0003), *APC* (the overall OR = 1.87, 95% CI = 1.11-3.14, p = 0.02), *p15* (the overall OR = 2.66, 95% CI = 1.25-5.64, p = 0.01) and* SFRP1* (the overall OR = 3.43, 95% CI = 1.19-9.88, p = 0.02). Our meta-analysis was unable to find any statistical significance between HBV-positive carcinoma tissues and HBV-negative carcinoma tissues for the methylation of the remaining seven genes, including *RUNX3*, *p14*, *WIF1*, *CDH1*, *PRDM2*, *SOCS1* and *MGMT*.

As shown in Table 2, the meta-analysis of *GSTP1* gene was involved with six studies between 84 HBV-positive adjacent tissues and 63 HBV-negative adjacent tissues. Our results revealed that the frequency of *GSTP1* gene methylation in HBV-positive adjacent tissues was significantly higher than HBV-negative adjacent tissues (the overall OR = 2.54, 95% CI = 1.04-6.21, p = 0.04). However, our meta-analysis was unable to find any statistical significance between HBV-positive adjacent tissues and HBV-negative adjacent tissues for the methylation of the remaining seven genes, including *p16*, *RASSF1A*, *APC*, *RUNX3*, *SOCS1*, *CDH1* and *SFRP1*.

As shown in Table 3, our meta-analysis showed statistical significance between HBV-positive carcinoma serums and HBV-negative carcinoma serums for the methylation of two genes, including *p16* (the overall OR = 2.51, 95% CI = 1.16-5.44, p = 0.02) and *APC* (the overall OR = 5.11, 95% CI = 2.09-12.52, p = 0.0004). However, our meta-analysis was unable to find any statistical significance between HBV-positive carcinoma serums and HBV-negative carcinoma serums for the methylation of the remaining *RASSF1A* gene.

In this meta-analysis, we selected various geographical populations to analyze any source of heterogeneity. We didn't find any significance in 13 methylated genes between HBV-positive carcinoma tissues and HBV-negative carcinoma tissues (Supplementary Table 2), eight methylated genes between HBV-positive adjacent tissues and HBV-negative adjacent tissues (Supplementary Table 3) and three methylated genes between HBV-positive carcinoma serums and HBV-negative carcinoma serums (Supplementary Table 4) in geographical populations. Our meta-analysis indicated that the heterogeneity was not contributed by geographical populations.

**Figure 3:**
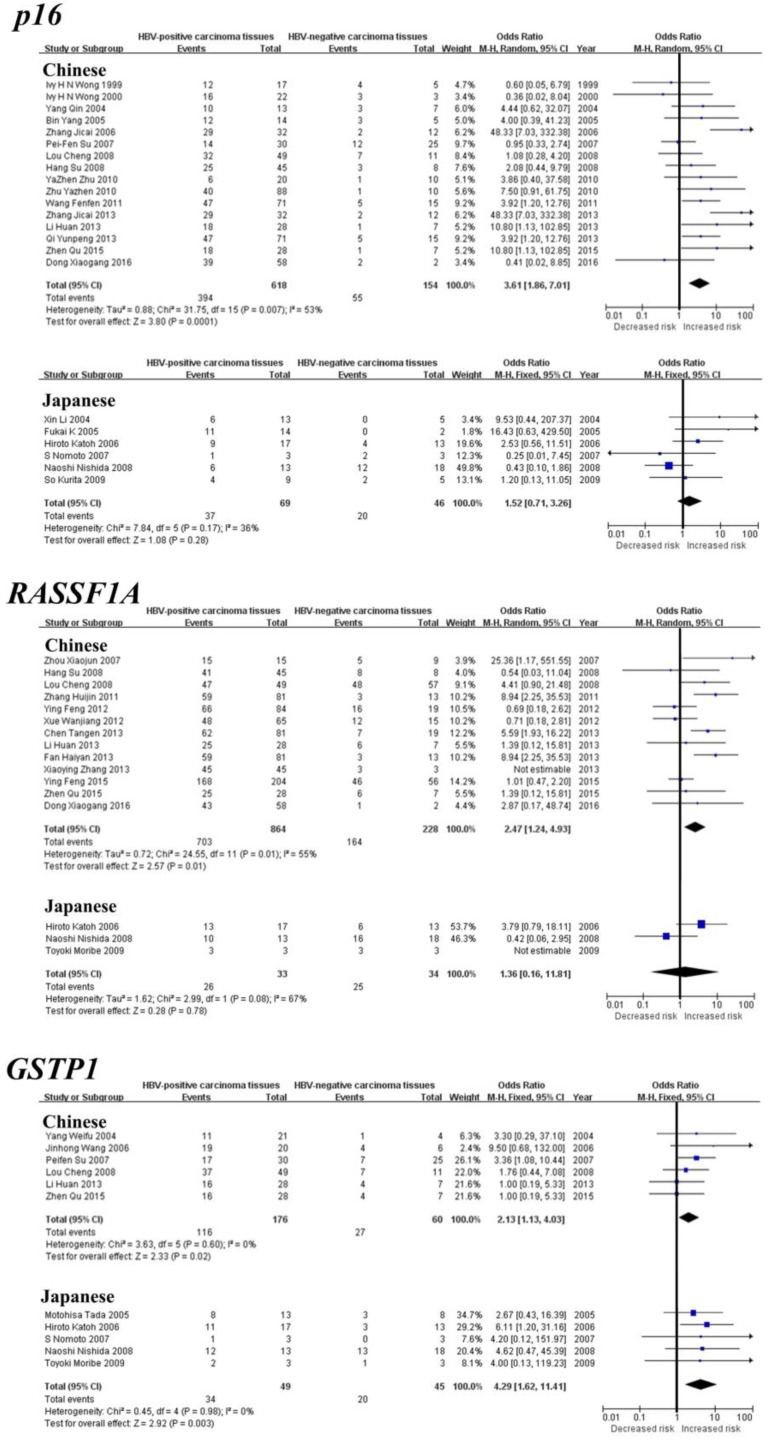
Forest plots of *p16*, *RASSF1A* and *GSTP1* methylation between HBV-positive carcinoma tissues and HBV-negative carcinoma tissues in HCC in the meta-analysis.

Subgroup meta-analysis by geographical populations was performed for *p16*, *RASSF1A*, *GSTP1*, *APC* and *RUNX3* between HBV-positive carcinoma tissues and HBV-negative carcinoma tissues. As shown in Figure 3, we found a statistical difference between HBV-positive carcinoma tissues and HBV-negative carcinoma tissues for *GSTP1* methylation in China from six studies (OR = 2.13, 95% CI = 1.13-4.03, I^2^ = 0%, p = 0.02) and in Japan from five studies (OR = 4.29, 95% CI = 1.62-11.41, I^2^ = 0%, p = 0.003). In addition, there was a significant geographical difference in the meta-analysis of *p16* methylation in China (OR = 3.61, 95% CI = 1.86-7.01, I^2^ = 53%, p = 0.0001), but not in Japan (OR = 1.52, 95% CI = 0.71-3.26, I^2^ = 36%, p = 0.28). A similar result was also found in *RASSF1A* (China: OR = 2.47, 95% CI = 1.24-4.93, I^2^ = 55%, p = 0.01; Japan: OR = 1.36, 95% CI = 0.16-11.81, I^2^ = 67%, p = 0.78). The subgroup meta-analysis of *APC* and *RUNX3* was unable to observe any significant result in each geographical population (Supplementary Figure 1).

## Discussion

Our meta-analysis included numerous studies that evaluated the contribution of HBV infection to DNA methylation in HCC. The meta-analysis focused on 13 genes between HBV-positive carcinoma tissues and HBV-negative carcinoma tissues, eight genes between HBV-positive adjacent tissues and HBV-negative adjacent tissues and three genes between HBV-positive carcinoma serums and HBV-negative carcinoma serums. In six genes (*p16*, *RASSF1A*, *GSTP1*, *APC*, *p15* and *SFRP1*), there was significant difference in the level of hypermethylation between HBV-positive carcinoma tissues and HBV-negative carcinoma tissues, revealing that HBV infection could induce methylation of these genes in HCC. One gene (*GSTP1*) hypermethylation showed significant difference between HBV-positive adjacent tissues and HBV-negative adjacent tissues, revealing that HBV infection could induce methylation of this gene in the early stage of hepatocarcinogenesis.

This meta-analysis showed that methylation of *GSTP1* gene in HBV-positive carcinoma tissues was significantly higher than in HBV-negative carcinoma tissues, and the same consequence was also found in adjacent tissues. This revealed that methylation of *GSTP1* gene could contribute to the whole process of the hepatocarcinogenesis of HBV-related HCC. In addition, methylation of *p16*, *RASSF1A*, *APC*, *p15* and *SFRP1* genes in HBV-positive carcinoma tissues was significantly higher than HBV-negative carcinoma tissues but not adjacent tissues, revealing that methylation of these five genes may play a significant role in the middle-late stage of the hepatocarcinogenesis process.

On the other hand, this meta-analysis showed that methylation of *RASSF1A* gene in HBV-positive carcinoma tissues was significantly higher than HBV-negative carcinoma tissues, while the same phenomenon was not found in carcinoma serums. The effects of this gene methylation in two sample types are different, due to the diversities of specificity and sensitivity in different sample types 25. Also, subgroup meta-analysis by geographical populations found that methylation of *GSTP1* was significant in both China and Japan. However, methylation of *p16* and *RASSF1A* genes was significant only in China, but not in Japan. The effects of these genes methylation are different, due to the diversities of hereditary in the different geographic regions 19, 26. In addition, the lack of association for these genes in Japanese population might be due to the small size of samples. For example, the size of carcinoma tissue samples in the meta-analysis of *p16* methylation in Chinese population was larger than the sample size of Japanese population, which might lead to the different associations.

Some studies showed that DNA methylation was associated with HBV-related HCC 21, 27-29. However, the mechanism of HBV infection in DNA methylation to induce hepatocarcinogenesis has not been clarified. Some studies found that hepatitis B virus X protein (HBx) could play a crucial role in epigenetic tumorigenesis of HBV-related HCC 30-32. HBx could increase the expressions of DNA-methyltransferase1 (DNMT1) and DNMT3b to induce hypermethylation of tumor suppressor genes 28, 33-35. DNA hypermethylation could silence the expressions of TSGs and lead to hepatocarcinogenesis. In this meta-analysis, methylation of some TSGs (*p16*, *RASSF1A*, *GSTP1*, *APC*, *p15* and *SFRP1*) could be induced by HBV infection, while other TSGs methylation could not be induced. We speculated that interactions of HBx and DNMTs of these TSGs caused the different effects. In the future, we need to explore the biological mechanisms of HBx-induced DNA methylation in HCC.

However, there were several limitations in this meta-analysis. Firstly, selection bias might exist because literatures only in English and Chinese were included in the meta-analysis. Secondly, this study focused on the genes with at least three independent studies and excluded those two studies within large size of samples. Thirdly, the main geographical populations of the meta-analysis were Chinese and Japanese. In the future, studies in other geographical populations were needed to explore the contribution of DNA methylation to HBV-related HCC.

In summary, this meta-analysis showed that DNA methylation and HBV infection were associated with the development of HCC. HBV infection could induce DNA methylation in HCC and DNA methylation contributed to the development of HBV-related HCC. Our results provided new clues for epigenetic therapy of HBV-related HCC.

## Supplementary Material

Supplementary figures and tables.

## Figures and Tables

**Table 1:** Characteristics of 13 genes methylation between HBV-positive carcinoma tissues and HBV-negative carcinoma tissues in HCC

Gene	Studies (n)	Overall OR (95% CI)	I^2^	P value	HBV-positive carcinoma tissues/HBV-negative carcinoma tissues
*p16*	24	2.87 [2.05, 4.01]	49%	<0.00001	708/224
*RASSF1A*	18	2.05 [1.44, 2.92]	46%	<0.0001	914/228
*GSTP1*	12	2.50 [1.52, 4.12]	0%	0.0003	234/129
*APC*	10	1.87 [1.11, 3.14]	0%	0.02	342/120
*RUNX3*	8	1.32 [0.81, 2.16]	0%	0.27	292/104
*p14*	6	1.75 [0.93, 3.29]	39%	0.08	191/93
*WIF1*	6	0.73 [0.43, 1.25]	0%	0.25	430/62
*CDH1*	6	1.01 [0.61, 1.67]	56%	0.96	255/111
*PRDM2*	6	1.61 [0.81, 3.20]	15%	0.17	156/51
*p15*	6	2.66 [1.25, 5.64]	0%	0.01	103/55
*SOCS1*	5	1.60 [0.75, 3.45]	8%	0.23	58/52
*SFRP1*	4	3.43 [1.19, 9.88]	0%	0.02	65/60
*MGMT*	3	1.46 [0.62, 3.45]	0%	0.39	114/41

**Table 2:** Characteristics of eight genes methylation between HBV-positive adjacent tissues and HBV-negative adjacent tissues in HCC

Gene	Studies (n)	Overall OR (95% CI)	I^2^	P value	HBV-positive adjacent tissues/HBV-negative adjacent tissues
*p16*	10	1.39 [0.71, 2.71]	0%	0.33	274/103
*GSTP1*	6	2.54 [1.04, 6.21]	0%	0.04	84/63
*RASSF1A*	3	0.94 [0.08, 11.22]	63%	0.96	63/29
*APC*	3	0.62 [0.07, 5.28]	0%	0.66	21/24
*RUNX3*	3	3.07 [0.40, 23.77]	0%	0.28	21/24
*SOCS1*	3	1.79 [0.20, 16.36]	72%	0.61	39/36
*CDH1*	3	0.91 [0.35, 2.36]	11%	0.85	78/44
*SFRP1*	3	1.86 [0.40, 8.62]	0%	0.43	40/55

**Table 3:** Characteristics of three genes methylation between HBV-positive carcinoma serums and HBV-negative carcinoma serums in HCC

Gene	Studies (n)	Overall OR (95% CI)	I^2^	P value	HBV-positive carcinoma serums/HBV-negative carcinoma serums
*p16*	10	2.51 [1.16, 5.44]	55%	0.02	460/122
*RASSF1A*	7	1.30 [0.76, 2.23]	0%	0.34	371/69
*APC*	3	5.11 [2.09, 12.52]	0%	0.0004	228/46

## Abbreviations

PLC: Primary liver cancer
HCC: Hepatocellular carcinoma
HBV: Hepatitis B virus
HBx: Hepatitis B virus X protein
CNKI: China National Knowledge Infrastructure
OR: Odds ratio
CI: Confidence interval
*RASSF1A*: Ras association domain family member1A
*GSTP1*: Glutathione S-transferase pi 1
*APC*: Adenomatous polyposis coli
*RUNX3*: Runt-related transcription factor 3
*WIF1*: WNT inhibitory factor 1
*CDH1*: E-cadherin
*PRDM2*: PR domain containing 2
*SOCS1*: Suppressor of the cytokine signalling 1
*SFRP1*: Secreted frizzled-related protein 1
*MGMT*: O6-methylguanine-DNA methyltransferase
DNMT1: DNA-methyltransferase 1
DNMT3b: DNA-methyltransferase 3b
TSGs: Tumor suppressor genes
