# Supplementary material for: Association between gene methylation and HBV infection in hepatocellular carcinoma: A meta-analysis
Source: J Cancer. 2019 Oct 20;10(25):6457–65. doi: 10.7150/jca.33005 (PMC6856736; doi:10.7150/jca.33005)

Supplementary Materials

Supplementary Document 1: PRISMA checklist.

Section/topic	#	Checklist item	Reported on page #
**TITLE**			
Title	1	Identify the report as a systematic review, meta-analysis, or both.	1
**ABSTRACT**			
Structured summary	2	Provide a structured summary including, as applicable: background; objectives; data sources; study eligibility criteria, participants, and interventions; study appraisal and synthesis methods; results; limitations; conclusions and implications of key findings; systematic review registration number.	2
**INTRODUCTION**			
Rationale	3	Describe the rationale for the review in the context of what is already known.	3
Objectives	4	Provide an explicit statement of questions being addressed with reference to participants, interventions, comparisons, outcomes, and study design (PICOS).	3
**METHODS**			
Protocol and registration	5	Indicate if a review protocol exists, if and where it can be accessed (e.g., Web address), and, if available, provide registration information including registration number.	4
Eligibility criteria	6	Specify study characteristics (e.g., PICOS, length of follow-up) and report characteristics (e.g., years considered, language, publication status) used as criteria for eligibility, giving rationale.	4
Information sources	7	Describe all information sources (e.g., databases with dates of coverage, contact with study authors to identify additional studies) in the search and date last searched.	4
Search	8	Present full electronic search strategy for at least one database, including any limits used, such that it could be repeated.	4
Study selection	9	State the process for selecting studies (i.e., screening, eligibility, included in systematic review, and, if applicable, included in the meta-analysis).	4
Data collection	10	Describe method of data extraction from reports (e.g., piloted	4

process		forms, independently, in duplicate) and any processes for obtaining and confirming data from investigators.	
Data items	11	List and define all variables for which data were sought (e.g., PICOS, funding sources) and any assumptions and simplifications made.	4
Risk of bias in individual studies	12	Describe methods used for assessing risk of bias of individual studies (including specification of whether this was done at the study or outcome level), and how this information is to be used in any data synthesis.	5
Summary measures	13	State the principal summary measures (e.g., risk ratio, difference in means).	5
Synthesis of results	14	Describe the methods of handling data and combining results of studies, if done, including measures of consistency (e.g., I^2) for each meta-analysis.	5
Section/topic	#	Checklist item	Reported on page #
Risk of bias across studies	15	Specify any assessment of risk of bias that may affect the cumulative evidence (e.g., publication bias, selective reporting within studies).	5
Additional analyses	16	Describe methods of additional analyses (e.g., sensitivity or subgroup analyses, meta-regression), if done, indicating which were pre-specified.	8-9
**RESULTS**			
Study selection	17	Give numbers of studies screened, assessed for eligibility, and included in the review, with reasons for exclusions at each stage, ideally with a flow diagram.	6
Study characteristics	18	For each study, present characteristics for which data were extracted (e.g., study size, PICOS, follow-up period) and provide the citations.	4
Risk of bias within studies	19	Present data on risk of bias of each study and, if available, any outcome level assessment (see item 12).	6-7, Table 1-3, Figure 2
Results of individual studies	20	For all outcomes considered (benefits or harms), present, for each study: (a) simple summary data for each intervention group (b) effect estimates and confidence intervals, ideally with a forest plot.	7-8, Table 1-3
Synthesis of results	21	Present results of each meta-analysis done, including confidence intervals and measures of consistency.	7-8, Table 1-3
Risk of bias across studies	22	Present results of any assessment of risk of bias across studies (see Item 15).	6-7, Table 1-3, Figure

			2
Additional analysis	23	Give results of additional analyses, if done (e.g., sensitivity or subgroup analyses, meta-regression [see Item 16]).	8-9
**DISCUSSION**			
Summary of evidence	24	Summarize the main findings including the strength of evidence for each main outcome; consider their relevance to key groups (e.g., healthcare providers, users, and policy makers).	10-11
Limitations	25	Discuss limitations at study and outcome level (e.g., risk of bias), and at review-level (e.g., incomplete retrieval of identified research, reporting bias).	11
Conclusions	26	Provide a general interpretation of the results in the context of other evidence, and implications for future research.	11-12
**FUNDING**			
Funding	27	Describe sources of funding for the systematic review and other support (e.g., supply of data); role of funders for the systematic review.	13

From: Moher D, Liberati A, Tetzlaff J, Altman DG, The PRISMA Group (2009). Preferred Reporting Items for Systematic Reviews and Meta-Analyses: The PRISMA Statement. PLoS Med 6(7): e1000097. doi:10.1371/journal.pmed1000097

For more information, visit: **www.prisma-statement.org**.

Supplementary Document 2: 69 eligible studies in this meta-analysis.

References

1. Wong IH, Lo YM, Zhang J, Liew CT, Ng MH, Wong N, Lai PB, Lau WY, Hjelm NM, Johnson PJ: Detection of aberrant p16 methylation in the plasma and serum of liver cancer patients. Cancer Res 1999, 59(1):71-3.
2. Wong IH, Lo YM, Yeo W, Lau WY, Johnson PJ: Frequent p15 promoter methylation in tumor and peripheral blood from hepatocellular carcinoma patients. Clin Cancer Res 2000, 6(9):3516-21.
3. Yang B, Guo M, Herman JG, Clark DP: Aberrant promoter methylation profiles of tumor suppressor genes in hepatocellular carcinoma. Am J Pathol 2003, 163(3):1101-7.
4. Qin Y, Liu JY, Li B, Sun ZL, Sun ZF: Association of low p16INK4a and p15INK4b mRNAs expression with their CpG islands methylation with human hepatocellular carcinogenesis. World J Gastroenterol 2004, 10(9):1276-80.
5. Li X, Hui AM, Sun L, Hasegawa K, Torzilli G, Minagawa M, Takayama T, Makuuchi M: p16INK4A hypermethylation is associated with hepatitis virus infection, age, and gender in hepatocellular carcinoma. Clin Cancer Res 2004, 10(22):7484-9.
6. Yang B, Gao YT, Du Z, Zhao L, Song WQ: Methylation-based molecular margin analysis in hepatocellular carcinoma. Biochem Biophys Res Commun 2005, 338(3):1353-8.
7. Fukai K, Yokosuka O, Imazeki F, Tada M, Mikata R, Miyazaki M, Ochiai T, Saisho H: Methylation status of p14ARF, p15INK4b, and p16INK4a genes in human hepatocellular carcinoma. Liver Int 2005, 25(6):1209-16.
8. Katoh H, Shibata T, Kokubu A, Ojima H, Fukayama M, Kanai Y, Hirohashi S: Epigenetic instability and chromosomal instability in hepatocellular carcinoma. Am J Pathol 2006, 168(4):1375-84.
9. Jicai Z, Zongtao Y, Jun L, Haiping L, Jianmin W, Lihua H: Persistent infection of hepatitis B virus is involved in high rate of p16 methylation in hepatocellular carcinoma. Mol Carcinog 2006, 45(7):530-6.
10. Su PF, Lee TC, Lin PJ, Lee PH, Jeng YM, Chen CH, Liang JD, Chiou LL, Huang GT, Lee HS: Differential DNA methylation associated with hepatitis B virus infection in hepatocellular carcinoma. Int J Cancer 2007, 121(6):1257-64.
11. Nomoto S, Kinoshita T, Kato K, Otani S, Kasuya H, Takeda S, Kanazumi N, Sugimoto H, Nakao A: Hypermethylation of multiple genes as clonal markers in multicentric hepatocellular carcinoma. Br J Cancer 2007, 97(9):1260-5.
12. Nishida N, Nagasaka T, Nishimura T, Ikai I, Boland CR, Goel A: Aberrant methylation of multiple tumor suppressor genes in aging liver, chronic hepatitis, and hepatocellular carcinoma. Hepatology 2008, 47(3):908-18.
13. Su H, Zhao J, Xiong Y, Xu T, Zhou F, Yuan Y, Zhang Y, Zhuang SM: Large-scale analysis of the genetic and epigenetic alterations in hepatocellular carcinoma from Southeast China. Mutat Res 2008, 641(1-2):27-35.
14. Kurita S, Ohkoshi S, Yano M, Yamazaki K, Suzuki K, Aoki YH, Matsuda Y, Wakai T, Shirai Y, Ichida T, Aoyagi Y: Progression of hypermethylation of the p16(INK4A) gene from normal liver to nontumorous liver and hepatocellular carcinoma: an evaluation using quantitative PCR analysis. Dig Dis Sci 2009, 54(1):80-8.

15. Feng Q, Stern JE, Hawes SE, Lu H, Jiang M, Kiviat NB: DNA methylation changes in normal liver tissues and hepatocellular carcinoma with different viral infection. Exp Mol Pathol 2010, 88(2):287-92.
16. Zhu YZ, Zhu R, Shi LG, Mao Y, Zheng GJ, Chen Q, Zhu HG: Hepatitis B virus X protein promotes hypermethylation of p16(INK4A) promoter through upregulation of DNA methyltransferases in hepatocarcinogenesis. Exp Mol Pathol 2010, 89(3):268-75.
17. Qu Z, Jiang Y, Li H, Yu DC, Ding YT: Detecting abnormal methylation of tumor suppressor genes GSTP1, P16, RIZ1, and RASSF1A in hepatocellular carcinoma and its clinical significance. Oncol Lett 2015, 10(4):2553-2558.
18. Lou C: The experimental research on DNA methylation profile in hepatocelluIar carcinoma. Tianjin Med Univ 2008.
19. Zhu YZ: Research on Hepatitis B Virus X Protein promoting hypermethylation of p16INK4A promoter. Fudan Univ 2010.
20. Wang FF: Correlation of aberrant methylation of p16, APC gene to MTHFR, TS genetic polymorphisms in hepatic carcinoma. Qingdao Univ 2011.
21. Qi YP, Wang FF, Cui LH, Song Y: Detection of methylation of p16 and APC gene in liver-cancer tissue and plasma and its clinical significance. Med J Qilu 2013, 28(3):189-91.
22. Li H: Study of aberrant methylation and clinical significance of suppressor gene in hepatocellular carcinoma. Soochow Univ 2013.
23. Zhang JC, Yu ZT, Lv J, Xie F, Gao B, Li HP: The relationgship between Hepatitis B Virus infection and Multigene methylation in hepatocellular carcinoma. J Clin Intern Med 2013, 30(3):163-5.
24. Dong XG, Guo WJ, Ding W: Detection of methylation of p16 and RASSF1A gene in plasma and tumor tissues from patientswith hepatocellular carcinoma and its clinical significance. Chin Med Biotechnol 2016, 11(3):252-258.
25. Moribe T, Iizuka N, Miura T, Kimura N, Tamatsukuri S, Ishitsuka H, Hamamoto Y, Sakamoto K, Tamesa T, Oka M: Methylation of multiple genes as molecular markers for diagnosis of a small, well-differentiated hepatocellular carcinoma. Int J Cancer 2009, 125(2):388-97.
26. Saelee P, Wongkham S, Chariyalertsak S, Petmitr S, Chuensumran U: RASSF1A promoter hypermethylation as a prognostic marker for hepatocellular carcinoma. Asian Pac J Cancer Prev 2010, 11(6):1677-81.
27. Feng Y, Xue WJ, Li P, Sha ZY, Huang H, Rui L, Li HX, Mao QS: RASSF1A hypermethylation is associated with aflatoxin B1 and polycyclic aromatic hydrocarbon exposure in hepatocellular carcinoma. Hepatogastroenterology 2012, 59(118):1883-8.
28. Zhang X, Li HM, Liu Z, Zhou G, Zhang Q, Zhang T, Zhang J, Zhang C: Loss of heterozygosity and methylation of multiple tumor suppressor genes on chromosome 3 in hepatocellular carcinoma. J Gastroenterol 2013, 48(1):132-43.
29. Feng Y, Li P, Liu Y, Sha Z, Feng L, Wang F, Mao Q, Xue W: The Association of Ala133Ser Polymorphism and Methylation in Ras Association Domain Family 1A Gene With Unfavorable Prognosis of Hepatocellular Carcinoma. Hepat Mon 2015, 15(10):e32145.
30. Araújo OC, Rosa AS, Fernandes A, Niel C, Villela-Nogueira CA, Pannain V, Araujo NM: RASSF1A and DOK1 Promoter Methylation Levels in Hepatocellular Carcinoma, Cirrhotic and Non-Cirrhotic Liver, and Correlation with Liver Cancer in Brazilian Patients. PLoS One 2016, 11(4): e0153796.

31. Zhou XJ, Qin L, Xue WJ, Liu JX, Tian LP, Qian HX: Inactivation of RASSF1A gene in hepatocellular carcinoma. Chin J Hepatobiliary Surg 2007, 13(5):351-2.
32. Zhang HJ: Prognostic significance of RASSFlA, APC, WIF-1 methylation in paraffin embedded tissues of HCC. Hebei Univ 2011.
33. Xue WJ, Feng Y, Li P, Mao QS, Guan HG, Qian HX: Correlation analysis of ras association domain family IA gene methylation and environmental factors to hepatocellular carcinoma. Chin J Exp Surg 2012, 29(4):650-2.
34. Fan HY, Zhang HJ, Guo ZJ, Li SM: Prognostic significance of RASSF1 A and WIF-1 methylation in the cancer tissues of patients with HCC. J Shandong Univ 2013, 51(5):89-93.
35. Chen TG, Li JG, Lin ZC: Promoter methylation of RASSFlA gene in hepatocellular carcinoma and its clinical significance. Chin J Gen Surg 2013, 28(4):300-3.
36. Tada M, Yokosuka O, Fukai K, Chiba T, Imazeki F, Tokuhisa T, Saisho H: Hypermethylation of NAD(P)H: quinone oxidoreductase 1 (NQO1) gene in human hepatocellular carcinoma. J Hepatol 2005, 42(4):511-9.
37. Wang J, Qin Y, Li B, Sun Z, Yang B: Detection of aberrant promoter methylation of GSTP1 in the tumor and serum of Chinese human primary hepatocellular carcinoma patients. Clin Biochem 2006, 39(4):344-8.
38. Yang WF: Detection aberrant promoter methylation of GSTP1 gene in hepatocellular carcinoma. Soochow Univ 2004.
39. Wu L, Qian YB, Zhu LX, Geng XP, Xiong QR: Promoter methylation and mRNA expression of SFRP1 and APC gene in hepatocellular carcinoma. Chin J Gen Surg 2008, 17(1):29-33.
40. Park WS, Cho YG, Kim CJ, Song JH, Lee YS, Kim SY, Nam SW, Lee SH, Yoo NJ, Lee JY: Hypermethylation of the RUNX3 gene in hepatocellular carcinoma. Exp Mol Med 2005, 37(4):276-81.
41. Zhang HY, Li K, He XB: Methylation status analysis of Runx3 gene in hepatocellular carcinoma and clinical significance. Clinical Focus 2009, 24(5):409-12.
42. Zhang HY, Liu J, Xie TB: Promoter hypermethylation of the Runx3 gene in hepatocellular carcinoma. Acta Med Univ Sci Technol Huazhong 2009, 38(1):23-6.
43. Jiang XJ, Li JG, Lin ZC: Relationship between promoter methylation and mRNA expression of RUNX3 gene in human primary hepatocellular carcinoma and its significance. Chin J Exp Surg 2011, 28(10):1640-2.
44. Li JG, Jiang XJ: Methylation of RUNX3 gene promoter in HCC and its significance. J Third Mil Med Univ 2012, 34(19):1933-5.
45. Wu FX, Chen J, Yang C, Cao J, Ou C, Zhao YG: p14ARF promoter methylation and its clinical correlation in hepatocellular carcinoma. J Guangxi Med Univ 2009, 26(2):218-20.
46. Zhang JX, Wang Y, Zhao YP, Zhang B, Li J, Huang J: p14ARF promoter methylation and its clinical correlation in primary liver cancer in the population of North China. J Clin Hepatol 2011, 27(10):1051-4.
47. Huang L, Li MX, Wang L, Li BK, Chen GH, He LR, Xu L, Yuan YF: Prognostic value of Wnt inhibitory factor-1 expression in hepatocellular carcinoma that is independent of gene methylation. Tumour Biol 2011, 32(1):233-40.
48. Liang H: Frequent epigenetic inactivation of Wnt inhibitory factor-1 in hepatocellular carcinoma. Sun Yatsen University 2008.
49. Ding Z, Qian YB, Xiong QR, Yu HZ, Li FY, Zhang C, Xu YC: Promoter methylation and

mRNA expression of DKK-3 gene in hepatocellular carcinoma. J Clin Med Pract 2009, 13(2):12-5.
50. Tong B: Methylation of WIF-1 gene and its expression in hepatocellular carcinoma. Tianjin Med Univ 2010.
51. Dou CY, Fan YC, Cao CJ, Yang Y, Wang K: Sera DNA methylation of CDH1, DNMT3b and ESR1 promoters as biomarker for the early diagnosis of hepatitis B virus-related hepatocellular carcinoma. Dig Dis Sci 2016, 61(4):1130-8.
52. Huang WQ, Yang WL, Chai XJ, Chen KF, Wei L, Li B, Qin Y: Aberrant promoter CpG islands methylation of E-cadherin in human primary hepatocellular carcinomas. Chin J Bases Clin General Surg 2011, 18(5):514-9.
53. Qian B, Zhu LX, Geng XP, Xiong QR, Qian YB, Li XM: Methylation of MGMT, DAPK, THBS1 and RIZ1 genes in hepatocellular carcinoma. Chin J Gen Surg 2005, 20(5):291-4.
54. Liu JY, Qin Y, Sun ZL, Sun ZF: Methylation of p15 and p16 gene 5' CpG island in human primary liver cancer. J Sichuan Univ 2002, 39:127-32.
55. Okochi O, Hibi K, Sakai M, Inoue S, Takeda S, Kaneko T, Nakao A: Methylation-mediated silencing of SOCS-1 gene in hepatocellular carcinoma derived from cirrhosis. Clin Cancer Res 2003, 9(14):5295-8.
56. Chu PY, Yeh CM, Hsu NC, Chang YS, Chang JG, Yeh KT: Epigenetic alteration of the SOCS1 gene in hepatocellular carcinoma. Swiss Med Wkly 2010, 140:w13065.
57. Saelee P, Chuensumran U, Wongkham S, Chariyalertsak S, Tiwawech D, Petmitr S: Hypermethylation of suppressor of cytokine signaling 1 in hepatocellular carcinoma patients. Asian Pac J Cancer Prev 2012, 13(7):3489-93.
58. Shih YL, Shyu RY, Hsieh CB, Lai HC, Liu KY, Chu TY, Lin YW: Promoter methylation of the secreted frizzled-related protein 1 gene SFRP1 is frequent in hepatocellular carcinoma. Cancer 2006, 107(3):579-90.
59. Su Q: Clinical research of the methylation status of 14-3-3 sigma gene and SFRP genes in hepatitis B virus-related hepatocellular carcinoma. Anhui Med Univ 2009.
60. Yang B: CDKN2A gene methylation and its clinical study in hepatocellular carcinoma. Nankai Univ 2006.
61. Zhang YJ, Wu HC, Shen J, Ahsan H, Tsai WY, Yang HI, Wang LY, Chen SY, Chen CJ, Santella RM: Predicting hepatocellular carcinoma by detection of aberrant promoter methylation in serum DNA. Clin Cancer Res 2007, 13(8):2378-84.
62. Lin Q, Tang YM, Chen LB, Wang J: Analysis of promoter hypermethylation of p16gene,AFP and CEA in sera from primary liver cancer patients. J Dalian Med Univ 2006, 28(4):290-2.
63. Zhang JC, Lv J, Li HP, Feng J, Wu JM, Hu LH: p16 promoter hypermethylation in the plasma DNA and its possible application in molecular diagnosis of hepatocellular carcinoma. Chin J Lab Med 2006, 29(10):895-898.
64. He QF, Yan D, Wang LX: Promoter hypermethylation of RASSF1A and p16 gene in plasma of patients with hepatocellular carcinoma. Chin J Public Health 2010, 26(7):819-21.
65. Yang JJ, Dang DM, Wang MQ, Ren CJ, Hui QY: The gene methylation of p16, Runx3 in patients with hepatocellular carcinoma. Modern Oncology 2013, 21(05):1081-4.
66. Qiu XQ, Chen G, Yu HP, Hu L: Detection of RASSF1A promoter hypermethylation in plasma of patients with primary hepatocellular carcinoma and its clinical significance. World Chin J Dig 2009, 17(1):90-93.

67. Fei BJ, Huang ZH, Hua D, Hu Y, Cheng ZH, Yu J: Clinical significance of methylation of plasma Ras·association domain family 1A gene in the molecular diagnosis of hepatocellular carcinoma. Tumor 2011, 31(8):742-7.
68. Gong HY, Liu ZG, Zhang HY: Detection of promoter hypermethylation of RASSF1A and CDH13 gene by nested methylation specific polymerase chain reaction in hepatocellular carcinoma patients. Life Science Res 2011, 15(1):56-60.
69. Zhao HJ, Li SM, Zhang HJ, Guo ZJ, Fan HY, Yang GR, Wang L, Zhang N: Diagnostic and prognostic value of RASSF1A and APC gene methylation in plasma of liver cancer patients. Chin J Cancer Prev Treat 2013, 20(1):53-7.

Supplementary Table 1: The basic characteristics of the included studies in the meta-analysis.

Gene	Author	Year	Geographical population	Sample type	Method	HBV-positive HCC	HBV-negative HCC
p16							
	Ivy H N Wong [1]	1999	Chinese	carcinoma tissue	MSP	17	5
	Ivy H N Wong [2]	2000	Chinese	carcinoma tissue	MSP	22	3
	Bin Yang [3]	2003	American	carcinoma tissue	MSP	9	24
	Yang Qin [4]	2004	Chinese	carcinoma tissue	MSP	13	7
	Xin Li [5]	2004	Japanese	carcinoma tissue	MSP	13	5
	Bin Yang [6]	2005	Chinese	carcinoma tissue	MSP	14	5
	Fukai K [7]	2005	Japanese	carcinoma tissue	MSP	14	2
	Hiroto Katoh [8]	2006	Japanese	carcinoma tissue	MSP	17	13
	Zhang Jicai [9]	2006	Chinese	carcinoma tissue	MSP	32	12
	Pei-Fen Su [10]	2007	Chinese	carcinoma tissue	MSP	30	25
	S Nomoto [11]	2007	Japanese	carcinoma tissue	MSP	3	3
	Naoshi Nishida [12]	2008	Japanese	carcinoma tissue	MSP	13	18
	Hang Su [13]	2008	Chinese	carcinoma tissue	MSP	45	8
	So Kurita [14]	2009	Japanese	carcinoma tissue	MSP	9	5
	Qinghua Feng [15]	2010	American	carcinoma tissue	MSP	12	0
	YaZhen Zhu [16]	2010	Chinese	carcinoma tissue	MSP	20	10
	Zhen Qu [17]	2015	Chinese	carcinoma tissue	MSP	28	7
	Lou Cheng [18]	2008	Chinese	carcinoma tissue	MSP	49	11
	Zhu Yazhen [19]	2010	Chinese	carcinoma tissue	MSP	88	10
	Wang Fenfen [20]	2011	Chinese	carcinoma tissue	MSP	71	15
	Qi Yunpeng [21]	2013	Chinese	carcinoma tissue	MSP	71	15
	Li Huan [22]	2013	Chinese	carcinoma tissue	MSP	28	7

	Zhang Jicai [23]	2013	Chinese	carcinoma tissue	MSP	32	12
	Dong Xiaogang [24]	2016	Chinese	carcinoma tissue	MSP	58	2
RASSF1A							
	Hiroto Katoh [8]	2006	Japanese	carcinoma tissue	MSP	17	13
	Naoshi Nishida [12]	2008	Japanese	carcinoma tissue	MSP	13	18
	Hang Su [13]	2008	Chinese	carcinoma tissue	MSP	45	8
	Toyoki Moribe [25]	2009	Japanese	carcinoma tissue	MSP	3	3
	Pensri Saelee [26]	2010	Thai	carcinoma tissue	MSP	13	10
	Ying Feng [27]	2012	Chinese	carcinoma tissue	MSP	84	19
	Xiaoying Zhang [28]	2013	Chinese	carcinoma tissue	MSP	45	3
	Zhen Qu [17]	2015	Chinese	carcinoma tissue	MSP	28	7
	Ying Feng [29]	2015	Chinese	carcinoma tissue	MSP	204	56
	Oscar C [30]	2016	Brazilian	carcinoma tissue	MSP	4	2
	Zhou Xiaojun [31]	2007	Chinese	carcinoma tissue	MSP	15	9
	Lou Cheng [18]	2008	Chinese	carcinoma tissue	MSP	49	11
	Zhang Huijin [32]	2011	Chinese	carcinoma tissue	MSP	81	13
	Xue Wanjiang [33]	2012	Chinese	carcinoma tissue	MSP	65	15
	Li Huan [22]	2013	Chinese	carcinoma tissue	MSP	28	7
	Fan Haiyan [34]	2013	Chinese	carcinoma tissue	MSP	81	13
	Chen Tangen [35]	2013	Chinese	carcinoma tissue	MSP	81	19
	Dong Xiaogang [24]	2016	Chinese	carcinoma tissue	MSP	58	2
GSTP1							
	Bin Yang [3]	2003	American	carcinoma tissue	MSP	9	24

	Motohisa Tada [36]	2005	Japanese	carcinoma tissue	MSP	13	8
	Jinhong Wang [37]	2006	Chinese	carcinoma tissue	MSP	20	6
	Hiroto Katoh [8]	2006	Japanese	carcinoma tissue	MSP	17	13
	Peifen Su [10]	2007	Chinese	carcinoma tissue	MSP	30	25
	S Nomoto [11]	2007	Japanese	carcinoma tissue	MSP	3	3
	Naoshi Nishida [12]	2008	Japanese	carcinoma tissue	MSP	13	18
	Toyoki Moribe [25]	2009	Japanese	carcinoma tissue	MSP	3	3
	Zhen Qu [17]	2015	Chinese	carcinoma tissue	MSP	28	7
	Yang Weifu [38]	2004	Chinese	carcinoma tissue	MSP	21	4
	Lou Cheng [18]	2008	Chinese	carcinoma tissue	MSP	49	11
	Li Huan [22]	2013	Chinese	carcinoma tissue	MSP	28	7
APC							
	Bin Yang [3]	2003	American	carcinoma tissue	MSP	9	24
	Hiroto Katoh [8]	2006	Japanese	carcinoma tissue	MSP	17	13
	S Nomoto [11]	2007	Japanese	carcinoma tissue	MSP	3	3
	Naoshi Nishida [12]	2008	Japanese	carcinoma tissue	MSP	13	18
	Toyoki Moribe [25]	2009	Japanese	carcinoma tissue	MSP	3	3
	Lou Cheng [18]	2008	Chinese	carcinoma tissue	MSP	49	11
	Wu Long [39]	2008	Chinese	carcinoma tissue	MSP	25	5
	Wang Fenfen [20]	2011	Chinese	carcinoma tissue	MSP	71	15
	Zhang Huijin [32]	2011	Chinese	carcinoma tissue	MSP	81	13
	Qi Yunpeng [21]	2013	Chinese	carcinoma tissue	MSP	71	15
RUNX3							
	Won Sang Park [40]	2005	Korean	carcinoma tissue	MSP	64	4

	S Nomoto [11]	2007	Japanese	carcinoma tissue	MSP	3	3
	Naoshi Nishida [12]	2008	Japanese	carcinoma tissue	MSP	13	18
	Toyoki Moribe [25]	2009	Japanese	carcinoma tissue	MSP	3	3
	Haiyuan Zhang [41]	2009	Chinese	carcinoma tissue	MSP	58	10
	Zhang Haiyuan [42]	2009	Chinese	carcinoma tissue	MSP	43	4
	Jiang Xiaojie [43]	2011	Chinese	carcinoma tissue	MSP	50	25
	Li Jianguo [44]	2012	Chinese	carcinoma tissue	MSP	58	37
p14							
	Bin Yang [3]	2003	American	carcinoma tissue	MSP	9	24
	Fukai K [7]	2005	Japanese	carcinoma tissue	MSP	14	2
	Peifen Su [10]	2007	Chinese	carcinoma tissue	MSP	30	25
	Wu Feixiang [45]	2009	Chinese	carcinoma tissue	MSP	36	14
	Zhang Junxia [46]	2011	Chinese	carcinoma tissue	MSP	70	16
	Zhang Jicai [23]	2013	Chinese	carcinoma tissue	MSP	32	12
WIF1							
	Liang Huang [47]	2011	Chinese	carcinoma tissue	MSP	94	11
	Liang Huang [48]	2008	Chinese	carcinoma tissue	MSP	101	12
	Ding Zhen [49]	2009	Chinese	carcinoma tissue	MSP	29	4
	Tong Bai [50]	2010	Chinese	carcinoma tissue	MSP	44	9
	Zhang Huijin [32]	2011	Chinese	carcinoma tissue	MSP	81	13
	Fan Haiyan [34]	2013	Chinese	carcinoma tissue	MSP	81	13
CDH1							
	Bin Yang [3]	2003	American	carcinoma tissue	MSP	9	24

	Hiroto Katoh [8]	2006	Japanese	carcinoma tissue	MSP	17	13
	Peifen Su [10]	2007	Chinese	carcinoma tissue	MSP	30	25
	Naoshi Nishida [12]	2008	Japanese	carcinoma tissue	MSP	13	18
	Chengyun Dou [51]	2015	Chinese	carcinoma tissue	MSP	153	30
	Huang Wenqing [52]	2011	Chinese	carcinoma tissue	MSP	33	1
PRDM2							
	S Nomoto [11]	2007	Japanese	carcinoma tissue	MSP	3	3
	Naoshi Nishida [12]	2008	Japanese	carcinoma tissue	MSP	13	18
	Zhen Qu [17]	2015	Chinese	carcinoma tissue	MSP	28	7
	Qian Bo [53]	2005	Chinese	carcinoma tissue	MSP	35	5
	Lou Cheng [18]	2008	Chinese	carcinoma tissue	MSP	49	11
	Li Huan [22]	2013	Chinese	carcinoma tissue	MSP	28	7
p15							
	Ivy H N Wong [2]	2000	Chinese	carcinoma tissue	MSP	22	3
	Bin Yang [3]	2003	American	carcinoma tissue	MSP	9	24
	Yang Qin [4]	2004	Chinese	carcinoma tissue	MSP	13	7
	Fukai K [7]	2005	Japanese	carcinoma tissue	MSP	14	2
	Liu Jianyu [54]	2002	Chinese	carcinoma tissue	MSP	13	7
	Zhang Jicai [23]	2013	Chinese	carcinoma tissue	MSP	32	12
SOCS1							
	Osamu Okochi [55]	2003	Japanese	carcinoma tissue	MSP	8	6
	S Nomoto [11]	2007	Japanese	carcinoma tissue	MSP	3	3
	Naoshi Nishida [12]	2008	Japanese	carcinoma tissue	MSP	13	18

	Peiyi Chu [56]	2010	Chinese	carcinoma tissue	MSP	21	15
	Pensri Saelee [57]	2012	Thai	carcinoma tissue	MSP	13	10
SFRP1							
	YuLueng Shih [58]	2006	Chinese	carcinoma tissue	MSP	33	11
	S Nomoto [11]	2007	Japanese	carcinoma tissue	MSP	3	3
	Wu Long [39]	2008	Chinese	carcinoma tissue	MSP	25	5
	Su Qian [59]	2009	Chinese	carcinoma tissue	MSP	4	41
MGMT							
	Peifen Su [10]	2007	Chinsee	carcinoma tissue	MSP	30	25
	Qian Bo [53]	2005	Chinese	carcinoma tissue	MSP	35	5
	Lou Cheng [18]	2008	Chinese	carcinoma tissue	MSP	49	11
p16							
	Bin Yang [6]	2005	Chinese	adjacent tissue	MSP	14	5
	Zhang Jicai [9]	2006	Chinese	adjacent tissue	MSP	32	12
	Pei-Fen Su [10]	2007	Chinese	adjacent tissue	MSP	30	25
	S Nomoto [11]	2007	Japanese	adjacent tissue	MSP	3	3
	Naoshi Nishida [12]	2008	Japanese	adjacent tissue	MSP	15	18
	Hang Su [13]	2008	Chinese	adjacent tissue	MSP	45	8
	YaZhen Zhu [16]	2010	Chinese	adjacent tissue	MSP	20	10
	Liu Jianyu [54]	2002	Chinese	adjacent tissue	MSP	13	7
	Bin Yang [60]	2006	Chinese	adjacent tissue	MSP	14	5
	Zhu Yazhen [19]	2010	Chinese	adjacent tissue	MSP	88	10

GSTP1							
	Motohisa Tada [36]	2005	Japanese	adjacent tissue	MSP	13	8
	Jinhong Wang [37]	2006	Chinese	adjacent tissue	MSP	20	6
	Peifen Su [10]	2007	Chinese	adjacent tissue	MSP	30	25
	S Nomoto [11]	2007	Japanese	adjacent tissue	MSP	3	3
	Naoshi Nishida [12]	2008	Japanese	adjacent tissue	MSP	15	18
	Toyoki Moribe [25]	2009	Japanese	adjacent tissue	MSP	3	3
RASSF1A							
	Naoshi Nishida [12]	2008	Japanese	adjacent tissue	MSP	15	18
	Hang Su [13]	2008	Chinese	adjacent tissue	MSP	45	8
	Toyoki Moribe [25]	2009	Japanese	adjacent tissue	MSP	3	3
APC							
	S Nomoto [11]	2007	Japanese	adjacent tissue	MSP	3	3
	Naoshi Nishida [12]	2008	Japanese	adjacent tissue	MSP	15	18
	Toyoki Moribe [25]	2009	Japanese	adjacent tissue	MSP	3	3
RUNX3							
	S Nomoto [11]	2007	Japanese	adjacent tissue	MSP	3	3
	Naoshi Nishida [12]	2008	Japanese	adjacent tissue	MSP	15	18
	Toyoki Moribe [25]	2009	Japanese	adjacent tissue	MSP	3	3
SOCS1							
	S Nomoto [11]	2007	Japanese	adjacent tissue	MSP	3	3
	Naoshi Nishida [12]	2008	Japanese	adjacent tissue	MSP	15	18

	Peiyi Chu [56]	2010	Chinese	adjacent tissue	MSP	21	15
CDH1							
	Peifen Su [10]	2007	Chinese	adjacent tissue	MSP	30	25
	Naoshi Nishida [12]	2008	Japanese	adjacent tissue	MSP	15	18
	Huang Wenqing [52]	2011	Chinese	adjacent tissue	MSP	33	1
SFRP1							
	YuLueng Shih [58]	2006	Chinese	adjacent tissue	MSP	33	11
	S Nomoto [11]	2007	Japanese	adjacent tissue	MSP	3	3
	Su Qian [59]	2009	Chinese	adjacent tissue	MSP	4	41
p16							
	Ivy H N Wong [1]	1999	Chinese	carcinoma serum	MSP	17	5
	Ivy H N Wong [2]	2000	Chinese	carcinoma serum	MSP	22	3
	Yujing Zhang [61]	2007	Chinese	carcinoma serum	MSP	25	14
	Lin Qing [62]	2006	Chinese	carcinoma serum	MSP	36	28
	Zhang Jicai [63]	2006	Chinese	carcinoma serum	MSP	32	12
	He Qingfang [64]	2010	Chinese	carcinoma serum	MSP	83	17
	Wang Fenfen [20]	2011	Chinese	carcinoma serum	MSP	71	15
	Qi Yunpeng [21]	2013	Chinese	carcinoma serum	MSP	71	15
	Yang Jianjun [65]	2013	Chinese	carcinoma serum	MSP	45	11
	Dong Xiaogang [24]	2016	Chinese	carcinoma serum	MSP	58	2

RASSF1A							
	Yujing Zhang [61]	2007	Chinese	carcinoma serum	MSP	25	14
	Xiaoqiang Qiu [66]	2009	Chinese	carcinoma serum	MSP	30	5
	He Qingfang [64]	2010	Chinese	carcinoma serum	MSP	83	17
	Fei Bojian [67]	2011	Chinese	carcinoma serum	MSP	61	11
	Gong huanyu [68]	2011	Chinese	carcinoma serum	MSP	28	4
	Zhao Huijin [69]	2013	Chinese	carcinoma serum	MSP	86	16
	Dong Xiaogang [24]	2016	Chinese	carcinoma serum	MSP	58	2
APC							
	Wang Fenfen [20]	2011	Chinese	carcinoma serum	MSP	71	15
	Qi Yunpeng [21]	2013	Chinese	carcinoma serum	MSP	71	15
	Zhao Huijin [69]	2013	Chinese	carcinoma serum	MSP	86	16

Abbreviation

MSP: Methylation Specific PCR.

References

1. Wong IH, Lo YM, Zhang J, Liew CT, Ng MH, Wong N, Lai PB, Lau WY, Hjelm NM, Johnson PJ: Detection of aberrant p16 methylation in the plasma and serum of liver cancer patients. Cancer Res 1999, 59(1):71-3.
2. Wong IH, Lo YM, Yeo W, Lau WY, Johnson PJ: Frequent p15 promoter methylation in tumor and peripheral blood from hepatocellular carcinoma patients. Clin Cancer Res 2000, 6(9):3516-21.
3. Yang B, Guo M, Herman JG, Clark DP: Aberrant promoter methylation profiles of tumor suppressor genes in hepatocellular carcinoma. Am J Pathol 2003, 163(3):1101-7.
4. Qin Y, Liu JY, Li B, Sun ZL, Sun ZF: Association of low p16INK4a and p15INK4b mRNAs expression with their CpG islands methylation with human

hepatocellular carcinogenesis. World J Gastroenterol 2004, 10(9):1276-80.

5. Li X, Hui AM, Sun L, Hasegawa K, Torzilli G, Minagawa M, Takayama T, Makuuchi M: p16INK4A hypermethylation is associated with hepatitis virus infection, age, and gender in hepatocellular carcinoma. Clin Cancer Res 2004, 10(22):7484-9.
6. Yang B, Gao YT, Du Z, Zhao L, Song WQ: Methylation-based molecular margin analysis in hepatocellular carcinoma. Biochem Biophys Res Commun 2005, 338(3):1353-8.
7. Fukai K, Yokosuka O, Imazeki F, Tada M, Mikata R, Miyazaki M, Ochiai T, Saisho H: Methylation status of p14ARF, p15INK4b, and p16INK4a genes in human hepatocellular carcinoma. Liver Int 2005, 25(6):1209-16.
8. Katoh H, Shibata T, Kokubu A, Ojima H, Fukayama M, Kanai Y, Hirohashi S: Epigenetic instability and chromosomal instability in hepatocellular carcinoma. Am J Pathol 2006, 168(4):1375-84.
9. Jicai Z, Zongtao Y, Jun L, Haiping L, Jianmin W, Lihua H: Persistent infection of hepatitis B virus is involved in high rate of p16 methylation in hepatocellular carcinoma. Mol Carcinog 2006, 45(7):530-6.
10. Su PF, Lee TC, Lin PJ, Lee PH, Jeng YM, Chen CH, Liang JD, Chiou LL, Huang GT, Lee HS: Differential DNA methylation associated with hepatitis B virus infection in hepatocellular carcinoma. Int J Cancer 2007, 121(6):1257-64.
11. Nomoto S, Kinoshita T, Kato K, Otani S, Kasuya H, Takeda S, Kanazumi N, Sugimoto H, Nakao A: Hypermethylation of multiple genes as clonal markers in multicentric hepatocellular carcinoma. Br J Cancer 2007, 97(9):1260-5.
12. Nishida N, Nagasaka T, Nishimura T, Ikai I, Boland CR, Goel A: Aberrant methylation of multiple tumor suppressor genes in aging liver, chronic hepatitis, and hepatocellular carcinoma. Hepatology 2008, 47(3):908-18.
13. Su H, Zhao J, Xiong Y, Xu T, Zhou F, Yuan Y, Zhang Y, Zhuang SM: Large-scale analysis of the genetic and epigenetic alterations in hepatocellular carcinoma from Southeast China. Mutat Res 2008, 641(1-2):27-35.
14. Kurita S, Ohkoshi S, Yano M, Yamazaki K, Suzuki K, Aoki YH, Matsuda Y, Wakai T, Shirai Y, Ichida T, Aoyagi Y: Progression of hypermethylation of the p16(INK4A) gene from normal liver to nontumorous liver and hepatocellular carcinoma: an evaluation using quantitative PCR analysis. Dig Dis Sci 2009, 54(1):80-8.
15. Feng Q, Stern JE, Hawes SE, Lu H, Jiang M, Kiviat NB: DNA methylation changes in normal liver tissues and hepatocellular carcinoma with different viral infection. Exp Mol Pathol 2010, 88(2):287-92.
16. Zhu YZ, Zhu R, Shi LG, Mao Y, Zheng GJ, Chen Q, Zhu HG: Hepatitis B virus X protein promotes hypermethylation of p16(INK4A) promoter through upregulation of DNA methyltransferases in hepatocarcinogenesis. Exp Mol Pathol 2010, 89(3):268-75.

17. Qu Z, Jiang Y, Li H, Yu DC, Ding YT: Detecting abnormal methylation of tumor suppressor genes GSTP1, P16, RIZ1, and RASSF1A in hepatocellular carcinoma and its clinical significance. Oncol Lett 2015, 10(4):2553-2558.
18. Lou C: The experimental research on DNA methylation profile in hepatocelluIar carcinoma. Tianjin Med Univ 2008.
19. Zhu YZ: Research on Hepatitis B Virus X Protein promoting hypermethylation of p16INK4A promoter. Fudan Univ 2010.
20. Wang FF: Correlation of aberrant methylation of p16, APC gene to MTHFR, TS genetic polymorphisms in hepatic carcinoma. Qingdao Univ 2011.
21. Qi YP, Wang FF, Cui LH, Song Y: Detection of methylation of p16 and APC gene in liver-cancer tissue and plasma and its clinical significance. Med J Qilu 2013, 28(3):189-91.
22. Li H: Study of aberrant methylation and clinical significance of suppressor gene in hepatocellular carcinoma. Soochow Univ 2013.
23. Zhang JC, Yu ZT, Lv J, Xie F, Gao B, Li HP: The relationgship between Hepatitis B Virus infection and Multigene methylation in hepatocellular carcinoma. J Clin Intern Med 2013, 30(3):163-5.
24. Dong XG, Guo WJ, Ding W: Detection of methylation of p16 and RASSF1A gene in plasma and tumor tissues from patientswith hepatocellular carcinoma and its clinical significance. Chin Med Biotechnol 2016, 11(3):252-258.
25. Moribe T, Iizuka N, Miura T, Kimura N, Tamatsukuri S, Ishitsuka H, Hamamoto Y, Sakamoto K, Tamesa T, Oka M: Methylation of multiple genes as molecular markers for diagnosis of a small, well-differentiated hepatocellular carcinoma. Int J Cancer 2009, 125(2):388-97.
26. Saelee P, Wongkham S, Chariyalertsak S, Petmitr S, Chuensumran U: RASSF1A promoter hypermethylation as a prognostic marker for hepatocellular carcinoma. Asian Pac J Cancer Prev 2010, 11(6):1677-81.
27. Feng Y, Xue WJ, Li P, Sha ZY, Huang H, Rui L, Li HX, Mao QS: RASSF1A hypermethylation is associated with aflatoxin B1 and polycyclic aromatic hydrocarbon exposure in hepatocellular carcinoma. Hepatogastroenterology 2012, 59(118):1883-8.
28. Zhang X, Li HM, Liu Z, Zhou G, Zhang Q, Zhang T, Zhang J, Zhang C: Loss of heterozygosity and methylation of multiple tumor suppressor genes on chromosome 3 in hepatocellular carcinoma. J Gastroenterol 2013, 48(1):132-43.
29. Feng Y, Li P, Liu Y, Sha Z, Feng L, Wang F, Mao Q, Xue W: The Association of Ala133Ser Polymorphism and Methylation in Ras Association Domain Family 1A Gene With Unfavorable Prognosis of Hepatocellular Carcinoma. Hepat Mon 2015, 15(10):e32145.
30. Araújo OC, Rosa AS, Fernandes A, Niel C, Villela-Nogueira CA, Pannain V, Araujo NM: RASSF1A and DOK1 Promoter Methylation Levels in Hepatocellular Carcinoma, Cirrhotic and Non-Cirrhotic Liver, and Correlation with Liver Cancer in Brazilian Patients. PLoS One 2016, 11(4): e0153796.
31. Zhou XJ, Qin L, Xue WJ, Liu JX, Tian LP, Qian HX: Inactivation of RASSF1A gene in hepatocellular carcinoma. Chin J Hepatobiliary Surg 2007, 13(5):351-2.
32. Zhang HJ: Prognostic significance of RASSFlA, APC, WIF-1 methylation in paraffin embedded tissues of HCC. Hebei Univ 2011.

33. Xue WJ, Feng Y, Li P, Mao QS, Guan HG, Qian HX: Correlation analysis of ras association domain family IA gene methylation and environmental factors to hepatocellular carcinoma. Chin J Exp Surg 2012, 29(4):650-2.
34. Fan HY, Zhang HJ, Guo ZJ, Li SM: Prognostic significance of RASSF1 A and WIF-1 methylation in the cancer tissues of patients with HCC. J Shandong Univ 2013, 51(5):89-93.
35. Chen TG, Li JG, Lin ZC: Promoter methylation of RASSFlA gene in hepatocellular carcinoma and its clinical significance. Chin J Gen Surg 2013, 28(4):300-3.
36. Tada M, Yokosuka O, Fukai K, Chiba T, Imazeki F, Tokuhisa T, Saisho H: Hypermethylation of NAD(P)H: quinone oxidoreductase 1 (NQO1) gene in human hepatocellular carcinoma. J Hepatol 2005, 42(4):511-9.
37. Wang J, Qin Y, Li B, Sun Z, Yang B: Detection of aberrant promoter methylation of GSTP1 in the tumor and serum of Chinese human primary hepatocellular carcinoma patients. Clin Biochem 2006, 39(4):344-8.
38. Yang WF: Detection aberrant promoter methylation of GSTP1 gene in hepatocellular carcinoma. Soochow Univ 2004.
39. Wu L, Qian YB, Zhu LX, Geng XP, Xiong QR: Promoter methylation and mRNA expression of SFRP1 and APC gene in hepatocellular carcinoma. Chin J Gen Surg 2008, 17(1):29-33.
40. Park WS, Cho YG, Kim CJ, Song JH, Lee YS, Kim SY, Nam SW, Lee SH, Yoo NJ, Lee JY: Hypermethylation of the RUNX3 gene in hepatocellular carcinoma. Exp Mol Med 2005, 37(4):276-81.
41. Zhang HY, Li K, He XB: Methylation status analysis of Runx3 gene in hepatocellular carcinoma and clinical significance. Clinical Focus 2009, 24(5):409-12.
42. Zhang HY, Liu J, Xie TB: Promoter hypermethylation of the Runx3 gene in hepatocellular carcinoma. Acta Med Univ Sci Technol Huazhong 2009, 38(1):23-6.
43. Jiang XJ, Li JG, Lin ZC: Relationship between promoter methylation and mRNA expression of RUNX3 gene in human primary hepatocellular carcinoma and its significance. Chin J Exp Surg 2011, 28(10):1640-2.
44. Li JG, Jiang XJ: Methylation of RUNX3 gene promoter in HCC and its significance. J Third Mil Med Univ 2012, 34(19):1933-5.
45. Wu FX, Chen J, Yang C, Cao J, Ou C, Zhao YG: p14ARF promoter methylation and its clinical correlation in hepatocellular carcinoma. J Guangxi Med Univ 2009, 26(2):218-20.
46. Zhang JX, Wang Y, Zhao YP, Zhang B, Li J, Huang J: p14ARF promoter methylation and its clinical correlation in primary liver cancer in the population of North China. J Clin Hepatol 2011, 27(10):1051-4.
47. Huang L, Li MX, Wang L, Li BK, Chen GH, He LR, Xu L, Yuan YF: Prognostic value of Wnt inhibitory factor-1 expression in hepatocellular carcinoma that is independent of gene methylation. Tumour Biol 2011, 32(1):233-40.
48. Liang H: Frequent epigenetic inactivation of Wnt inhibitory factor-1 in hepatocellular carcinoma. Sun Yatsen University 2008.

49. Ding Z, Qian YB, Xiong QR, Yu HZ, Li FY, Zhang C, Xu YC: Promoter methylation and mRNA expression of DKK-3 gene in hepatocellular carcinoma. J Clin Med Pract 2009, 13(2):12-5.
50. Tong B: Methylation of WIF-1 gene and its expression in hepatocellular carcinoma. Tianjin Med Univ 2010.
51. Dou CY, Fan YC, Cao CJ, Yang Y, Wang K: Sera DNA methylation of CDH1, DNMT3b and ESR1 promoters as biomarker for the early diagnosis of hepatitis B virus-related hepatocellular carcinoma. Dig Dis Sci 2016, 61(4):1130-8.
52. Huang WQ, Yang WL, Chai XJ, Chen KF, Wei L, Li B, Qin Y: Aberrant promoter CpG islands methylation of E-cadherin in human primary hepatocellular carcinomas. Chin J Bases Clin General Surg 2011, 18(5):514-9.
53. Qian B, Zhu LX, Geng XP, Xiong QR, Qian YB, Li XM: Methylation of MGMT, DAPK, THBS1 and RIZ1 genes in hepatocellular carcinoma. Chin J Gen Surg 2005, 20(5):291-4.
54. Liu JY, Qin Y, Sun ZL, Sun ZF: Methylation of p15 and p16 gene 5' CpG island in human primary liver cancer. J Sichuan Univ 2002, 39:127-32.
55. Okochi O, Hibi K, Sakai M, Inoue S, Takeda S, Kaneko T, Nakao A: Methylation-mediated silencing of SOCS-1 gene in hepatocellular carcinoma derived from cirrhosis. Clin Cancer Res 2003, 9(14):5295-8.
56. Chu PY, Yeh CM, Hsu NC, Chang YS, Chang JG, Yeh KT: Epigenetic alteration of the SOCS1 gene in hepatocellular carcinoma. Swiss Med Wkly 2010, 140:w13065.
57. Saelee P, Chuensumran U, Wongkham S, Chariyalertsak S, Tiwawech D, Petmitr S: Hypermethylation of suppressor of cytokine signaling 1 in hepatocellular carcinoma patients. Asian Pac J Cancer Prev 2012, 13(7):3489-93.
58. Shih YL, Shyu RY, Hsieh CB, Lai HC, Liu KY, Chu TY, Lin YW: Promoter methylation of the secreted frizzled-related protein 1 gene SFRP1 is frequent in hepatocellular carcinoma. Cancer 2006, 107(3):579-90.
59. Su Q: Clinical research of the methylation status of 14-3-3 sigma gene and SFRP genes in hepatitis B virus-related hepatocellular carcinoma. Anhui Med Univ 2009.
60. Yang B: CDKN2A gene methylation and its clinical study in hepatocellular carcinoma. Nankai Univ 2006.
61. Zhang YJ, Wu HC, Shen J, Ahsan H, Tsai WY, Yang HI, Wang LY, Chen SY, Chen CJ, Santella RM: Predicting hepatocellular carcinoma by detection of aberrant promoter methylation in serum DNA. Clin Cancer Res 2007, 13(8):2378-84.
62. Lin Q, Tang YM, Chen LB, Wang J: Analysis of promoter hypermethylation of p16gene,AFP and CEA in sera from primary liver cancer patients. J Dalian Med Univ 2006, 28(4):290-2.
63. Zhang JC, Lv J, Li HP, Feng J, Wu JM, Hu LH: p16 promoter hypermethylation in the plasma DNA and its possible application in molecular diagnosis of

hepatocellular carcinoma. Chin J Lab Med 2006, 29(10):895-898.
64. He QF, Yan D, Wang LX: Promoter hypermethylation of RASSF1A and p16 gene in plasma of patients with hepatocellular carcinoma. Chin J Public Health 2010, 26(7):819-21.
65. Yang JJ, Dang DM, Wang MQ, Ren CJ, Hui QY: The gene methylation of p16, Runx3 in patients with hepatocellular carcinoma. Modern Oncology 2013, 21(05):1081-4.
66. Qiu XQ, Chen G, Yu HP, Hu L: Detection of RASSF1A promoter hypermethylation in plasma of patients with primary hepatocellular carcinoma and its clinical significance. World Chin J Dig 2009, 17(1):90-93.
67. Fei BJ, Huang ZH, Hua D, Hu Y, Cheng ZH, Yu J: Clinical significance of methylation of plasma Ras·association domain family 1A gene in the molecular diagnosis of hepatocellular carcinoma. Tumor 2011, 31(8):742-7.
68. Gong HY, Liu ZG, Zhang HY: Detection of promoter hypermethylation of RASSF1A and CDH13 gene by nested methylation specific polymerase chain reaction in hepatocellular carcinoma patients. Life Science Res 2011, 15(1):56-60.
69. Zhao HJ, Li SM, Zhang HJ, Guo ZJ, Fan HY, Yang GR, Wang L, Zhang N: Diagnostic and prognostic value of RASSF1A and APC gene methylation in plasma of liver cancer patients. Chin J Cancer Prev Treat 2013, 20(1):53-7.

Supplementary Table 2: Analysis of heterogeneity sources of 13 genes methylation between HBV-positive carcinoma tissues and HBV-negative carcinoma tissues in HCC in geographical populations.

Gene	Geographical population	Studies (n)	Coefficient	95% CI	P value
p16	China	16	-0.8607089	[-2.240385, 0.5189674]	0.209
	Japan	6	—	—	—
	America	2	-1.270956	[-4.303588, 1.761675]	0.394
RASSF1A	China	13	-1.610805	[-6.113554, 2.891943]	0.451
	Japan	3	-1.114868	[-5.950196, 3.72046]	0.625
	Thailand	1	-1.860752	[-7.469659, 3.748155]	0.484
	Brazil	1	—	—	—
GSTP1	China	6	-0.3695204	[-2.302005, 1.562964]	0.676
	Japan	5	-1.061634	[-3.170589, 1.047322]	0.284
	America	1	—	—	—
APC	China	5	-0.1584815	[-1.788317, 1.471354]	0.825
	Japan	4	—	—	—
	America	1	-0.4311435	[-2.806241, 1.943954]	0.681
RUNX3	China	4	0.0165502	[-1.765576, 1.798677]	0.982
	Japan	3	—	—	—
	Korea	1	-0.5228616	[-3.968048, 2.922325]	0.713
p14	China	4	—	—	—
	Japan	1	—	—	—
	America	1	1.455163	[-4.659908, 7.570233]	0.504
WIF1	China	6	—	—	—
CDH1	China	3	-0.4265984	[-3.87634, 3.023143]	0.72
	Japan	2	—	—	—
	America	1	-1.406721	[-6.14395, 3.330509]	0.414
PRDM2	China	4	0.6768346	[-2.008149, 3.361818]	0.523
	Japan	2	—	—	—
p15	China	4	-0.4680645	[-6.066942, 5.130813]	0.807
	Japan	1	—	—	—
	America	1	-0.901902	[-6.943279, 5.139475]	0.667
SOCS1	Japan	3	1.372043	[-4.381798 7.125883]	0.413
	China	1	1.450589	[-4.895154, 7.796331]	0.429
	Thailand	1	—	—	—
SFRP1	China	3	1.49404	[-6.79241, 9.780489]	0.519
	Japan	1	—	—	—
MGMT	China	3	—	—	—

Analysis of heterogeneity sources of *p16* in Japan, *RASSF1A* in Brazil, *GSTP1* in

America, *APC* in Japan, *RUNX3* in Japan, *p14* in China and Japan, *WIF1* in China, *CDH1* in Japan, *PRDM2* in Japan, *p15* in Japan, *SOCS1* in Thailand, *SFRP1* in Japan and *MGMT* in China was not applicable, because the data of these genes methylation were insufficient.

Supplementary Table 3: Analysis of heterogeneity sources of eight genes methylation between HBV-positive adjacent tissues and HBV-negative adjacent tissues in HCC in geographical populations.

Gene	Geographical population	Studies (n)	Coefficient	95% CI	P value
p16	China	8	—	—	—
	Japan	2	1.343732	[-1.666668, 4.354132]	0.326
GSTP1	Japan	4	1.025441	[-3.056455, 5.107338]	0.393
	China	2	—	—	—
RASSF1A	Japan	2	—	—	—
	China	1	—	—	—
APC	Japan	3	—	—	—
RUNX3	Japan	3	—	—	—
SOCS1	Japan	2	—	—	—
	China	1	—	—	—
CDH1	China	2	—	—	—
	Japan	1	—	—	—
SFRP1	China	2	—	—	—
	Japan	1	—	—	—

Analysis of heterogeneity sources of *p16* in China, *GSTP1* in China, *RASSF1A* in Japan and China, *APC* in Japan, *RUNX3* in Japan, *SOCS1* in Japan and China, *CDH1* in China and Japan, and *SFRP1* in China and Japan was not applicable, because the data of these genes methylation were insufficient.

Supplementary Table 4: Analysis of heterogeneity sources of three genes methylation between HBV-positive carcinoma serums and HBV-negative carcinoma serums in HCC in geographical populations.

Gene	Geographical population	Studies (n)	Coefficient	95% CI	P value
p16	China	10	—	—	—
RASSF1A	China	7	—	—	—
APC	China	3	—	—	—

Analysis of heterogeneity sources of *p16* in China, *RASSF1A* in China and *APC* in China was not applicable, because the data of these genes methylation were insufficient.

Supplementary Figure 1: Forest plots of *APC* and *RUNX3* methylation between HBV-positive carcinoma tissues and HBV-negative carcinoma tissues in HCC in the meta-analysis.

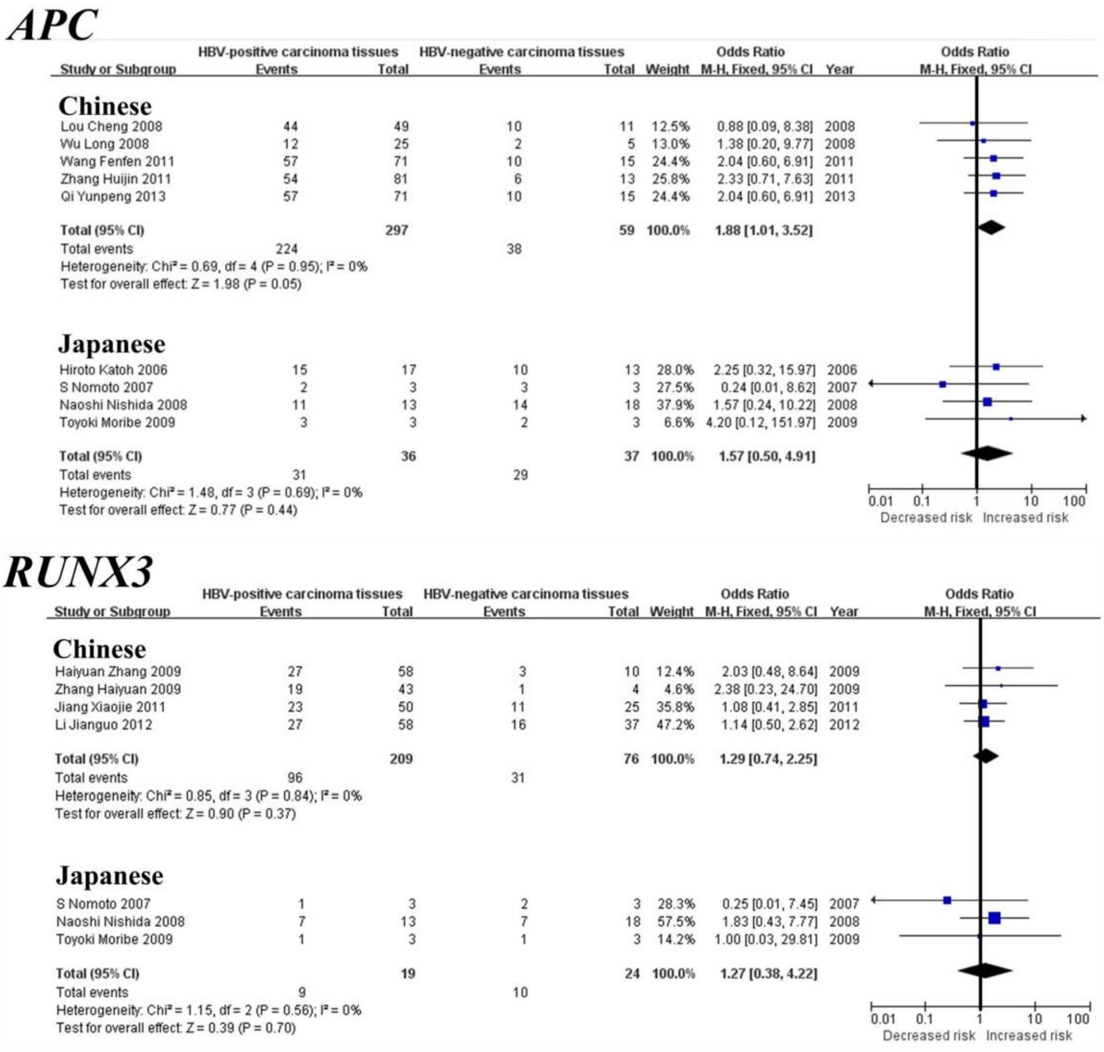

Supplement: Supplementary file 1 — Supplementary figures and tables. [file jcav10p6457s1.pdf]
